# Mechanistic Studies of Gypenosides in Microglial State Transition and its Implications in Depression-Like Behaviors: Role of TLR4/MyD88/NF-κB Signaling

**DOI:** 10.3389/fphar.2022.838261

**Published:** 2022-03-15

**Authors:** Li-Hua Cao, Yuan-Yuan Zhao, Ming Bai, David Geliebter, Jan Geliebter, Raj Tiwari, Hong-Juan He, Zhen-zhen Wang, Xing-Yuan Jia, Jin Li, Xiu-Min Li, Ming-San Miao

**Affiliations:** ^1^ Academy of Chinese Medical Sciences, Henan University of Chinese Medicine, Zhengzhou, China; ^2^ School of Pharmacy, Henan University of Chinese Medicine, Zhengzhou, China; ^3^ Beersheba Functional Medicine, Beersheba, Israel; ^4^ Department of Pathology, Microbiology and Immuology, New York Medical College, Valhalla, NY, United States; ^5^ Department of Otolaryngology, New York Medical College, Valhalla, NY, United States; ^6^ Department of Pharmacy, Henan Province Hospital of Traditional Chinese Medicine, Zhengzhou, China; ^7^ Department of Neurology, New York Medical College, Westchester Medical Center, Valhalla, NY, United States

**Keywords:** gypenosides, depression-like behaviors, TLR4/MyD88/NF-κB signaling, microglial state transition, microglial phenotypes

## Abstract

Depression is a prevalent psychiatric disorder. Microglial state transition has been found in many neurological disorders including depression. Gypenosides (Gypenosides I-LXXVIII, Gps) are saponin extracts isolated from the traditional Chinese herb *Gynostemma pentaphyllum* (Thunb.) Makino that exert anti-inflammatory and neuroprotective activities and regulate depression-like behaviors. However, its effect on microglial state transition in depression remains unknown. We aimed to evaluate the potential relationship between Gps and TLR4/MyD88/NF-κB signaling in microglial state transition *in vitro* and *in vivo*. First, BV-2 cells (microglial cell line) were exposed to lipopolysaccharides (LPS) and treated with 10 or 5 μg/ml Gps. Second, the chronic unpredictable mild stress (CUMS)-induced depression mouse model was used to investigate the antidepressant-like behaviors effects of Gps (100 or 50 mg/kg). We determined depression-like behaviors using the open-field test (OFT), forced swim test (FST), and sucrose preference test (SPT). Proteins and inflammatory factors in the TLR4/MyD88/NF-κB signaling pathway and the different microglial reaction states markers were subsequently conducted using enzyme-linked immunosorbent assay, immunocytochemistry, immunofluorescence, qPCR, or Western blotting analyses to evaluate the anti-inflammatory and antidepressant properties of Gps and the underlying molecular mechanisms. We found that Gps regulated the microglial cell line state transition in LPS-exposed BV-2 cells, as evidenced by the significantly decreased expression of inflammatory parameters iNOS, IL-1*β*, IL-6, and TNF-α and significantly promoted anti-inflammatory microglial phenotypes markers CD206 (Mrc1) and IL-10. More importantly, Gps protected against the loss of monoamine neurotransmitters and depression-like behavior in a mouse model of depression, which was accompanied by a regulation of the microglial state transition. Mechanistically, Gps inhibited TLR4/MyD88/NF-κB signaling, which reduced the release of downstream inflammatory cytokines (IL-1β, IL-6, and TNF-α) and promoted microglial phenotype transition, which all together contributed to the antidepressant effect. Our results suggest that Gps prevents depression-like behaviors by regulating the microglial state transition and inhibiting the TLR4/MyD88/NF-κB signaling pathway. Thus, Gps could be a promising therapeutic strategy to prevent and treat depression-like behaviors and other psychiatric disorders.

## 1 Introduction

Depression is an affective mental disorder characterized by chronic, recurrent, and life-threatening symptoms that has become a serious global health problem (Lim et al., 2018; Malhi and Mann, 2018). Patients suffering from depression are particularly likely to present with depressed mood, anhedonia, feelings of worthlessness or guilt, and suicidal ideation. The World Health Organization ranks depression as the third leading cause of the global burden of disease and predicts that it will rank first by 2030 (WHO, 2008). Due to the complex biological mechanisms of depression, numerous systems involved, and the fact that the exact mechanisms are not fully understood, research on antidepressants is a worldwide focus, but progress is hindered (Harmer et al., 2017; Tomlinson et al., 2018). Seeking novel approaches and new drug targets to treat depression has become an urgent unmet need.

Furthermore, the number of potential initial stressors that have been associated with depression is manifold and include adverse childhood experiences such as abuse, neglect, and household dysfunction (Tsehay et al., 2020); asthma (Frieri et al., 2015); autoimmune diseases, such as Hashimoto’s disease (Giynas Ayhan et al., 2014) and rheumatoid arthritis (Nerurkar et al., 2019); cancer (Szelei & Döme, 2020); dietary factors, such as high omega-6: omega-3 ratio (Hoge et al., 2019), soft drinks (Zhang et al., 2019), and ultra-processed food (Gómez-Donoso et al., 2020); emotional factors, such as financial stress (Price et al., 2002), marital status (Yan et al., 2011), and social isolation during COVID-19 pandemic (Robb et al., 2020); and environmental chemicals, such as certain heavy metals, phthalates, and polyaromatic hydrocarbons (Shiue, 2015). It is noteworthy that many of these stressors have the potential to be comorbid with each other, for instance adverse childhood experiences and autoimmune diseases (Dube et al., 2009). Given the numerous potential initial stressors—including the ones not mentioned in the preceding, non-exhaustive list—and the vast number of people experiencing depression symptoms, addressing a process that encompasses many of the stressors should be of interest to researchers and health practitioners in many fields, as well as their patients.

Relevant data from *in vitro* and *in vivo* experiments point to increased systemic inflammation and central nervous system (CNS) inflammation in depression disorders (Berk et al., 2013; Passos et al., 2015; Rahimian et al., 2021). As the innate immune cells of the CNS, microglia are the main members of the first line of immune defense of the CNS and play a central role in the immune and inflammatory response of the CNS. Over the last few decades, a number of studies have shown that the microglial over-reactivity to stress is involved in the pathological process of depression (Franklin et al., 2018; Klawonn et al., 2021). Recent studies suggest that microglia possess potent regenerative and immunoregulatory capacities (Ginhoux and Prinz, 2015), and are endowed with spectacular plasticity, allowing them to acquire multiple phenotypes and thereby fulfill numerous functions in health and disease (Stratoulias et al., 2019; Hammond et al., 2019; Benmamar-Badel, et al., 2020). Microglia might form a microglial community (Hammond et al., 2019; Stratoulias et al., 2019), a tremendous shift from the classical (“activated” or “M1”-phenotype)/alternative (“alternatively activated” or “M2”-phenotype) classification still used a few years ago (Kalkman and Feuerbach, 2016; Duan et al., 2020). For example, a number of studies have found that pro-inflammatory microglial phenotypes secrete proinflammatory cytokines consisting of tumor necrosis factor-α (TNF-α), interleukin (IL)-1β, IL-6, and iNOS, which lead to dysfunction of the neurotrophic system (Park et al., 2015; Zusso et al., 2019). In contrast, a neuroprotective microglial phenotype, contributes to antagonizing inflammation-induced damage by enhancing the expression of different mediators, such as IL-10 and TGF-β (Li et al., 2021; Spiteri et al., 2022). In addition, the dark microglia, a recently described phenotype rarely observed in the brain under steady state conditions, becomes abundant during nonhomeostatic conditions such as chronic stress, aging, and so on (Bisht et al., 2016; Stratoulias et al., 2019).

Growing evidence indicates that the change in microglia state may play an important role in controlling the balance between the generation and regression of CNS inflammation (Zhang et al., 2018; Lau, et al., 2021). Microglial state transition was reported in a chronic unpredictable mild stress (CUMS)-induced depression model, GRb1 treatment alleviated depressive-like behaviors in chronic mild stress (CMS)-exposed mice via inducing pro-neurogenic phenotype of microglia (Zhang et al., 2021). β-hydroxybutyrate can reduce CNS inflammation, accompanied by a shift in microglial profile toward anti-inflammatory phenotypes, in CNS inflammation models induced by LPS or CUMS (Huang et al., 2018). Research shows the presence of an increase in IL-6 and TNF-α levels in major depressive disorder (MDD) patients cerebrospinal fluid (CSF), and brain parenchyma, in the context of a possible increased microglial reaction (Enache et al., 2019). Unfortunately, a growing body of evidence from human autopsy found that abnormalities of the microglial states and functions have been observed in the brains of patients who committed suicide after depression (Yang et al., 2020; Brisch et al., 2021). These studies suggest that regulating the microglial state transition seems particularly important in the cause and treatment of depression. Nevertheless, the mechanisms underlying such changes have been only partially delineated. To regulate the phenotypic transformation of microglia and prevent and treat depression, understanding the mechanism of microglial state transition and finding effective treatments is an urgent unmet need (Stein et al., 2018). Notably, evidence points to the TLR4-MyD88/NF-κB signaling pathway as playing a key role in the immune response by affecting microglial state, leading to increased chronic low-grade inflammation and depression-like behaviors (Yang et al., 2019; Zhao et al., 2020).

The emerging role of CNS inflammation in the pathogenesis of depression has become a useful target for antidepressant drug discovery (Ma et al., 2017). A novel approach in the development of drugs to prevent and treat depression comes from the use of herbs (Liu et al., 2015). Gypenosides (Gypenosides I-LXXVIII, Gps) are a main functional component isolated from Gynostemma [*Gynostemma pentaphyllum* (Thunb.) Makino]. Research suggests that Gps have high oral availability and many kinds of pharmacological effects, such as anti-inflammatory and immunomodulatory effects, plus improving learning and memory ability. In 1995, it was reported that Gps combined with amitriptyline can treat depressive psychosis clinically (Zeng and Li, 1995). The latest research shows that Gps can improve mouse depression-like behavior by modulating neuroinflammatory pathways, including the hippocampal NF-κB pathway (Dong et al., 2018) and the BDNF-ERK/Akt signaling pathway (Mu et al., 2016). However, hippocampal microglial state transition has yet to be explored to determine the antidepressant mechanism of Gps.

To explore whether Gps could affect microglial state and to detail its potential mechanism, we established a model of depression through 5 weeks of CUMS and LPS-exposed microglial cell line (BV-2 cells). We investigated the mechanism of action of Gps as it affects depression-like behavior in association with TLR4/MyD88/NF-κB signaling and microglial state transition.

## 2 Materials and Methods

### 2.1 Cell Culture and Treatments

BV-2 murine microglial cell line cells were purchased from Procell Life Science and Technology Co., Ltd, cultured in DMEM (HyClone, United States) supplemented with 10% fetal bovine serum (Gibco, United States), and maintained in a 5% CO_2_ incubator at 37°C. The experiment was divided into control and three independent treatment groups: LPS (50 μg/ml) group, Gps-H (10 μg/ml) + LPS (50 μg/ml) group, and Gps-L (5 μg/ml) + LPS (50 μg/ml) group.

BV-2 cells (2 × 10^4^) were cultured in 6-well dishes 12 h before being exposed to LPS (50 μg/ml), Gps-H + LPS, or Gps-L + LPS. After 48 h, the culture supernatant and BV-2 cells were collected and stored at −80°C until further use.

### 2.2 Cellular Viability

The effect of Gps on cell viability was evaluated by the Cell Counting Kit-8 (CCK-8) (Dojindo, Japan) assay according to the manufacturer’s instructions. In brief, BV-2 cells (1 × 10^4^ cells/well) were seeded in a 96-well dish. After complete adherence to the walls, the cells were exposed to Gps (20, 10, 5, 2.5, and 0 μg/ml) for 24 or 48 h. The medium was removed, and the cells were incubated with 10% CCK-8 for 4 h. Absorbance was recorded at 450 nm with a microplate reader.

### 2.3 Cellular Immunofluorescence

BV-2 cells (2 × 10^4^) were cultured on coverslips and then incubated with LPS (50 μg/ml) or Gps for 48 h. Cells were then fixed in 4% paraformaldehyde for 30 min and treated with 0.1% Triton X-100 for 20 min, followed by blocking with 5% BSA (bovine serum albumin) for 30 min at room temperature. Cells were incubated with anti-iNOS antibody (ab283655, Abcam, Cambridge, MA, United States) or anti-CD206 antibody (#24595, Cell Signaling, United States) overnight for 4°C. After three washings with ice-cold PBS, the cells were incubated with a secondary antibody [Cy3 conjugated Goat Anti-Rabbit IgG (H + L), GB21303, Servicebio, China] for 1 h at room temperature. After three washings, the nuclei were counterstained with DAPI (4’,6-diamidino-2-phenylindole) for 10 min at 37°C. Different microglial state markers were observed using a fluorescence microscope [Olympus, Tokyo, Japan, numerical aperture (NA = 1.4)] and photographed at × 400 magnification. The mean fluorescent intensity was measured by ImageJ software.

### 2.4 Mice

Six-week-old specific pathogen-free male C57BL/6J mice were purchased from Beijing Vital River Laboratory Animal Technology Co., Ltd. (Beijing, China). All animals were housed five per cage in plastic cages with soft bedding and supplied with standard laboratory conditions of food and water *ad libitum*, 22 ± 2°C, a 12 h light-dark cycle (lights on at 08:00), and relative humidity 50–60% unless otherwise specified. All experiments were conducted in accordance with the guidelines of the Animal Care and Use Committee of Henan University of Chinese Medicine. Every effort was made to minimize the number and suffering of animals used.

### 2.5 Animal Model and Treatment Protocols

**FIGURE 1:**
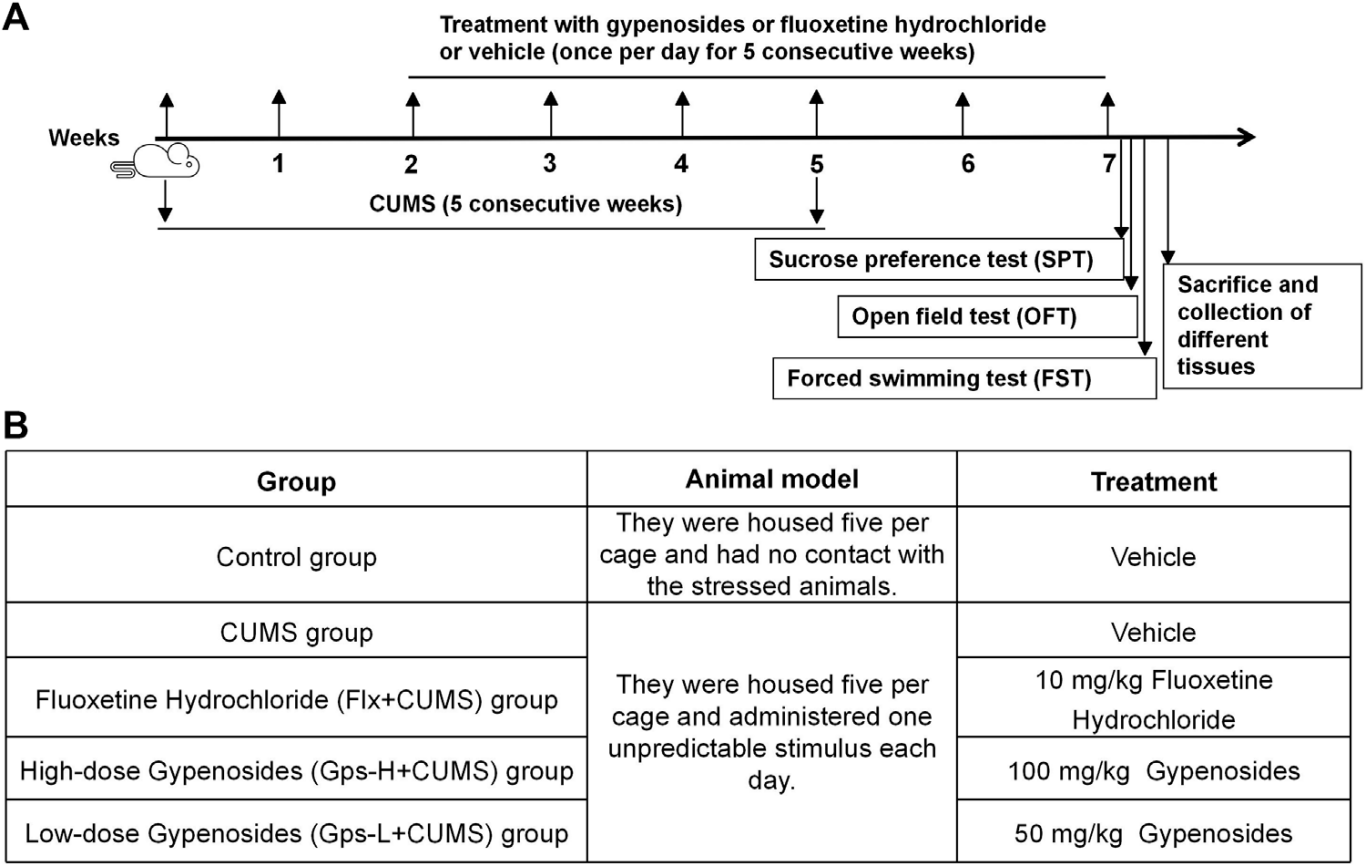
Schematic representation of the experimental design **(A)** and overview of the groups **(B)**.

After an accommodation period of 1 week, mice were randomly divided into five equal groups (*n* = 5 in each group), including the control group, CUMS group, fluoxetine hydrochloride (Flx + CUMS) group, high-dose gypenoside (Gps-H + CUMS) group, and low-dose gypenoside (Gps-L + CUMS) group. The mice were subjected to different stressors for 5 weeks except the control group. From the third week, groups of mice were intragastrically administered Flx (10 mg/kg), Gps-H (100 mg/kg), Gps-L (50 mg/kg), or a vehicle once per day for five consecutive weeks. CUMS stimuli were continued until the fifth week. A schematic for the treatment and CUMS stimulus timeline is depicted in Figure 1.

At the end of the treatment, behavioral tests were performed. Then, blood was collected using cardiac puncture under anesthesia with inhalational isoflurane (Xu et al.,. 2020a). Mice were sacrificed and subsequently, brains were rapidly dissected and washed with ice-cold saline. Then, each brain was divided into two parts across the sagittal plane. One part of the brain was fixed with 10% (v/v) formalin for 24 h. The other part of the brain was homogenized in ice-cold physiological saline to prepare a 10% homogenate. Serum was prepared by centrifuging at 10,000 rpm for 15 min at 4°C after standing for 30 min at room temperature. After sacrifice, serum, brain homogenate, and brain tissues were immediately isolated and stored at −80°C until further use.

#### 2.5.1 Chronic Unpredictable Mild Stress

The CUMS procedure was performed as described below (Table 1) (Fahim et al., 2019; Xu et al., 2020b; Yan et al., 2021). One stressor was applied each day. To prevent habituation and to ensure the unpredictability of the stressors, all stressors were randomly scheduled over a 7 days period and were repeated throughout 5 weeks. During this experiment, the control group mice were left undisturbed in the home cages.

**TABLE 1:** Chronic unpredictable mild stress (CUMS) procedure.

Stressor	Details
Food and water deprivation	Mice were subjected to 24 h of food and water deprivation. Food and water were provided immediately after the end of the fasting period
Continuous illumination	Continuous illumination for 24 h
Wet sawdust bedding	200 ml water in 100 g sawdust bedding for 24 h. After the stress, mice were removed from the tank and towel dried before being placed back in the home cage
Heat environment	Mice were placed in 45°C heat stress for 5 min, after the stress, they were placed back in home cage
Cold water swimming	Mice were placed for 5 min in a cylindrical clear plastic tank filled with 4°C cold water. After the swim, they were removed from the tank and towel dried before being placed back in home cage
Tail nipping	Tail pinch 1 cm from the beginning of the tail for 5 min
Physical restrain	Confinement in a tube for 1 h

#### 2.5.2 Sucrose Preference Test

The anhedonic condition of the mice was measured using the sucrose preference test (SPT) as previously described to assess depression levels. This protocol includes a two-part plan: adaptation and the preference test. During this experiment, the animals were singly housed. Specifically, during adaptation, the mice were exposed to two regular bottles (1% sucrose solution) for 24 h. Then, one bottle of sucrose solution was replaced with regular water for 24 h. Mice were subsequently deprived of food and water for 24 h. For the preference test, the animals were given 12 continuous hours of exposure to one bottle with sucrose solution and one with regular water. The volumes of each tube were recorded before and after the test. SPT was performed at night. The experimenters were blind to their identity of the samples.

The sucrose preference (SP) value was calculated as SP (%) = sucrose intake (vol)/[sucrose intake (vol) + water intake (vol)] × 100%.

#### 2.5.3 Open Field Test

Locomotor activity was evaluated using the open field test (OFT). The test was carried out in an open field apparatus with dimensions of 50 cm length × 50 cm wide × 40 cm high. During the test, a quiet experimental environment was maintained. At the beginning of the test, the mice were placed individually in the center of the open field apparatus and permitted free exploration for 5 min. After adaptation, the distance each mouse moved was recorded during the next 5 min. The open field apparatus was cleaned with 75% ethanol before evaluation of the next mouse. The observers were blind to group assignment of each mouse.

#### 2.5.4 Forced Swimming Test

The forced swimming test (FST) protocol was performed according to a previously described methodology, with minor modifications (Cao et al., 2019). In brief, mice were placed into a hyaline cylinder with dimensions of 15 cm diameter × 45 cm height and filled with 20 cm of water (22 ± 2°C) depth. All mice were individually placed in the water and forced to swim for 5 min. After an initial 2-min adaptation phase, video recordings (SuperFst forced FST software, XR-XQ202, Shanghai Xinruan Information technology Co., Ltd) were used for the immobility time during the final 4 min. The water was replaced after each trial. The observers were blind to group assignment of each mouse.

### 2.6 Enzyme-Linked Immunosorbent Assay

Measurements of serum and cell culture supernatant TNF-α, IL-6, and IL-1β levels were performed using a mouse TNF-α and IL-6 enzyme-linked immunosorbent assay (ELISA) development kit (Mabtech, Nacka, Sweden, 3511-1A-6, 3361-1A-6) and a mouse IL-1 beta (IL-1β) ELISA kit (Abcam, ab100704), respectively. The brain monoamine neurotransmitter levels were measured by a serotonin ELISA kit (Enzo Biochem Inc, United States, ADI-900-175), dopamine ELISA kit (Enzo Biochem Inc, United States, ENZ-KIT188-0001), and noradrenaline ELISA kit (Enzo Biochem Inc, United States, ASB-OKEH02565) according to the protocol of the manufacturer.

### 2.7 mRNA Expression Analysis

Total RNA of BV-2 cells and the brain (hippocampus) were isolated by TRIzol Reagent (Ambion, United States). The PrimeScriptTM RT reagent kit with gDNA Eraser (TAKARA, Japan) was used for reverse transcription, and the cDNA was stored at −20°C. qRT–PCR was performed in an Applied Biosystems 7500 Fast Real-Time PCR System (Thermo Fisher Scientific, United States) using a SYBR Premix Ex TaqTM II kit (TAKARA, Japan). Relative gene expression in relation to the reference gene (Actb) was calculated using the 2^-ΔΔCT^ method. Primers for TNF-α, IL-1β, IL-6, IL-10, CD206, and iNOS were commercially purchased from Sangon Biotech (Shanghai, China). Primers for IL-10 (NC_000,067.7) were 5′- GCT​CTT​ACT​GAC​TGG​CAT​GAG -3′ (forward) and 5′- CGC​AGC​TCT​AGG​AGC​ATG​TG -3′ (reverse). Primers for TNF-α (NC_000,083.7) were 5′- GAC​GTG​GAA​CTG​GCA​GAA​GAG -3′ (forward) and 5′- TTG​GTG​GTT​TGT​GAG​TGT​GAG -3′ (reverse). Primers for IL-1β (NC_000,068.8) were 5′- GCA​ACT​GTT​CCT​GAA​CTC​AAC​T -3′ (forward) and 5′- ATC​TTT​TGG​GGT​CCG​TCA​ACT -3′ (reverse). Primers for CD206 (NC_000,068.8) were 5′- CTC​TGT​TCA​GCT​ATT​GGA​CGC -3′ (forward) and 5′- CGG​AAT​TTC​TGG​GAT​TCA​GCT​TC -3′ (reverse). Primers for iNOS (NC_000,077.7) were 5′- GGA​GTG​ACG​GCA​AAC​ATG​ACT -3′ (forward) and 5′- TCG​ATG​CAC​AAC​TGG​GTG​AAC -3′ (reverse). Primers for IL-6 (NC_000,071.7) were 5′- TCT​ATA​CCA​CTT​CAC​AAG​TCG​GA -3′ (forward) and 5′- GAA​TTG​CCA​TTG​CAC​AAC​TCT​TT -3′ (reverse). Primers for GAPDH (NC_000072.7) were 5′-AGGTCGGTGTGAACGGATTTG-3′ (forward) and 5′-TGTAGACCATGTAGTTGAGGTCA-3′ (reverse).

### 2.8 Immunohistochemical and Immunofluorescence Analysis

After sacrifice, the brains were collected, fixed with 10% formalin for 24 h, dehydrated by incubations in different concentration of alcohol, and then cleared with xylene. Afterwards, brains were embedded in paraffin at 56°C in a hot air oven for 24 h. Coronal brain sections were processed for paraffin embedding, and 4 μm samples were sectioned (Cao et al., 2019).

Immunohistochemistry: The brain sections were treated with 0.02% Triton X-100 (Sigma, United States) for 20 min, and then incubated with blocking buffer (2% BSA in PBS) for 1 h. After washing with PBS, the sections were soaked in 0.3% PBST for 10 min, then blocked and incubated with anti-5-HT1A antibodies (ab85615, Abcam, United States) overnight. The sections were subsequently washed, and incubated with a secondary antibody (BS13278, Bioworld, United States) for 1 h at room temperature after which they were then stained with DBA (Thermo Fisher, United States). The sections were counterstained, dehydrated, and then examined under a light microscope (Olympus, Tokyo, Japan, NA = 1.4) to determine the expression of 5-HT1A in the hypothalamus, and photographed at × 400 magnification. Positive staining showed different degrees of yellow or pale brown, and the area percentage (Area%) was measured by ImageJ software.

Immunofluorescence: The brain sections were treated with 0.1% Triton X-100 for 20 min, followed by blocking with 5% BSA (bovine serum albumin) for 30 min at room temperature. Then, the samples were incubated with anti-CD206 antibody (AF2535-SP, R and D Systems, United States) and anti-iNOS antibody (ab283655, Abcam, Cambridge, MA, United States) overnight for 4°C. After three washings, the samples were incubated with Cy3 conjugated Goat Anti-mouse IgG (GB21303, Servicebio, China) and Alexa Fluor 488 anti-rabbit IgG (GB25303, Servicebio, China) for 50 min at room temperature. After washing with PBS again, the nuclei were counterstained with DAPI (4’,6-diamidino-2-phenylindole) for 10 min at 37°C. Different microglial state markers was observed using a fluorescence microscope (Olympus, Tokyo, Japan, NA = 1.4) and photographed at × 400 magnification. The mean fluorescent intensity was measured by ImageJ software.

### 2.9 Western Blot Analysis

The protein levels of iNOS, CD206, TLR4, MyD88, and NF-κB p65 were analyzed using western blotting methodology, as described previously (Xu et al.,. 2020a). The hippocampus and BV-2 cell line were isolated and lysed in RIPA lysis buffer (EpiZyme, Shanghai, China) and quantified by the BCA Protein Kit (23,227, Thermo Fisher, United States). The samples were diluted with the 5 × loading buffer, and processed at 100°C for 10 min. Equal amounts of protein were loaded and separated by SDS-polyacrylamide gel electrophoresis (PAGE) and transferred to polyvinylidene difluoride (PVDF) membranes (Millipore, United States). After blocking with 5% BSA (bovine serum albumin) for 1 h at room temperature, membranes were incubated with primary antibodies overnight at 4°C. After washing, secondary antibody was added for 1 h at room temperature, and detected by the enhanced chemiluminescence technique. Primary antibodies were as follows: TLR4 (ab13556, Abcam, United States), MyD88 (ab28763,Abcam, United States), NF-κB p65 (ab16502, Abcam, United States), and iNOS (ab283655, Abways, China) were purchased from Abcam, Cambridge, MA, United States, CD206 (YT5640, Immunaway, United States) and GAPDH (P04406, Abways, China). The secondary antibody was anti-rabbit IgG (H&L)-HRP (BS13278, Bioworld, United States).

### 2.10 Statistical Analysis

All the results were performed in at least a triplicate fashion and experimental data were expressed as the mean ± SEM. All data were analyzed with GraphPad Prism eight software (version 8, GraphPad Software, Inc, San Diego, CA, United States). After removal of outliers, the program ran the data to determine if the data were normally distributed or not. If the data were not normally distributed, a non-parametric test was applied. Statistical significance was determined using one-way ANOVA followed by Dunnett’s multiple comparisons test. The results were considered statistically significant when *p* < 0.05. All experiments were carried out in a blinded manner; experimenters were blind to identity of the samples.

## 3 Results

### 3.1 Gps Regulates Microglial Cell Line State Transition in LPS-Exposed BV-2 Cells

**FIGURE 2:**
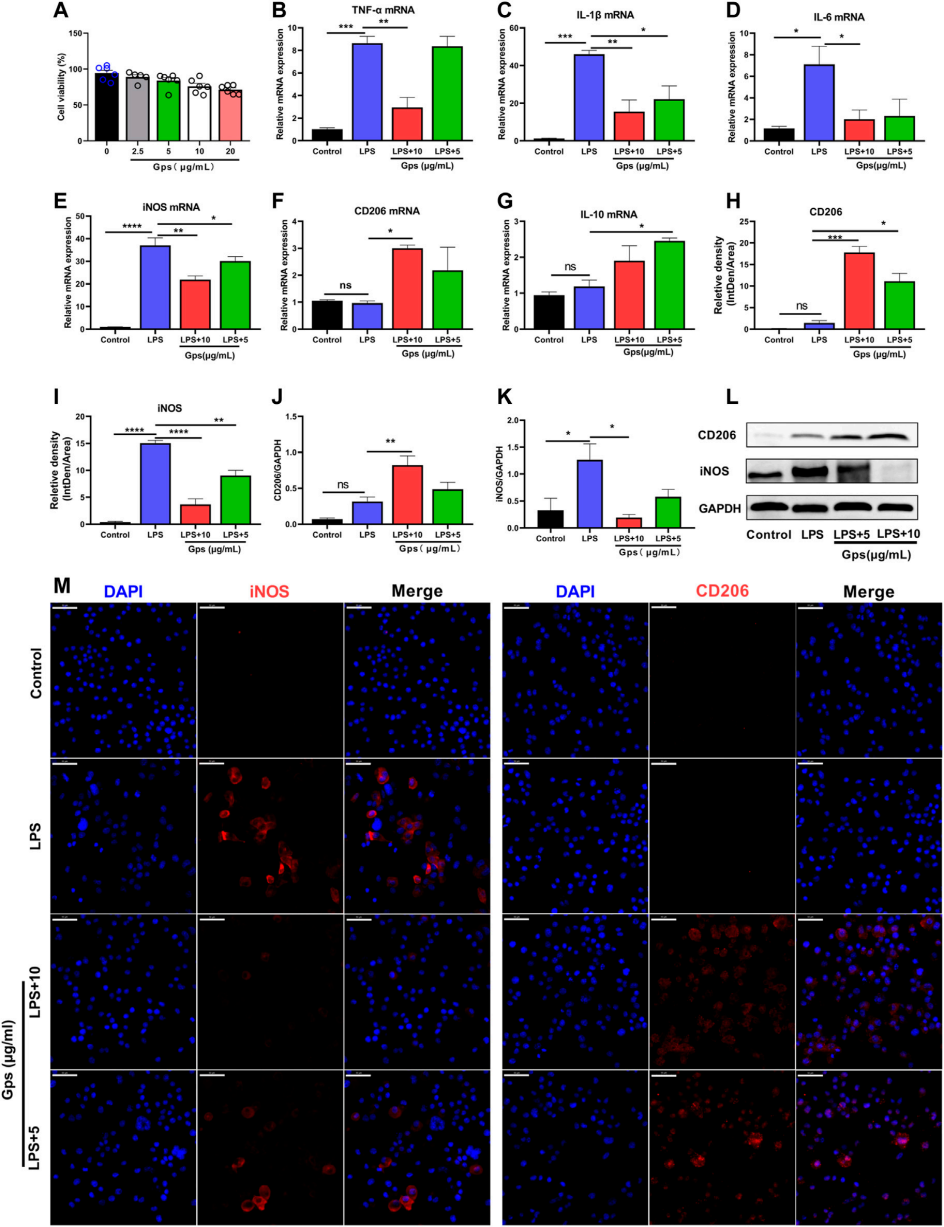
Gps regulates microglial cell line state transition in LPS-exposed BV-2 cells. **(A)** BV-2 cells (1 × 10^5^ cells/well) were seeded in 96-well culture plates, and exposed to Gps at concentrations of 0, 2.5, 5, 10, and 20 μg/ml. After 48 h, cell viability was assessed using a CCK-8 assay. The OD450 values of cell viability were shown on the y-axis. *n* = 6 for each groups from 2 sets of experiments. **(B–G)** The expression of TNF-03B1, IL-1β, IL-6, iNOS, CD206, and IL-10 mRNA, respectively, in control (untreated), LPS (50 μg/ml), and LPS (50 μg/ml) and Gps (10 μg/ml and 5 μg/ml, respectively) BV-2 cells were determined using qPCR. N = 3 from triplicate culture for each group. **(H,I)** The relative fluorescence intensity of iNOS and CD206 in the different groups of BV-2 cells. The values are expressed by IntDen/Area on the y-axis for different groups on the x-axis. *n* = 3 from triplicate culture for each group. **(J,K)**. Statistical results show that LPS increased the expression of iNOS protein *in vitro*, while Gps inhibited the effects and promoted the expression of protein CD206. *n* = 3 from triplicate culture for each group. **(L)**. Representative immunoreactive bands showing the iNOS and CD206 proteins in the LPS (50 μg/ml) and Gps (10 μg/ml or 5 μg/ml, respectively), LPS (50 μg/ml), and control BV-2 cells. M. Representative immunofluorescence picture of iNOS (red) and CD206 (red) in control (untreated), LPS (50 μg/ml), and LPS (50 μg/ml) and Gps (10 μg/ml or 5 μg/ml, respectively) BV-2 cells (× 400 magnification). Scale bar indication 50 µm. The results were expressed by mean ± SEM. *, *p* < 0.05; **, *p* < 0.01; ***, *p* < 0.001; ****, *p* < 0.0001 vs. LPS.

To determine whether Gps had an effect on inflammation and microglial state transition, we evaluated the viability of BV-2 cells treated with Gps. After 24 h, CCK-8 assays revealed that no cytotoxic effect was observed at concentrations of less than 10 μg/ml (data not shown). We used 10 and 5 μg/ml as Gps concentrations for all the subsequent experiments performed in this study (Figure 2A). Then, the effect of Gps (10 and 5 μg/ml) on LPS-exposed BV-2 cell state transition was assessed. We found that LPS increased the mRNA expression of the inflammatory parameters TNF-α, IL-1β, IL-6, and iNOS (Figures 2B–E,K,L) but not the mRNA expression of the anti-inflammatory phenotype markers CD206 and IL-10 (Figures 2F,G). Interestingly, Gps significantly ameliorated this effect and promoted the expression of IL-10 and CD206 mRNA (Figures 2F,G,J,L), emphasizing the effect of Gps treatment on the microglial state transition *in vitro*. To prove the role of Gps, we conducted an immunofluorescence experiment. Further observation found that Gps inhibited the number of iNOS + cells and promoted CD206 + cells in LPS-exposed BV-2 cells, which are favoring the anti-inflammatory microglial state, which are different states of microglial markers. respectively (Figures 2H,I,M).

### 3.2 Gps Regulates Microglial Cell Line State Transition by Inhibiting the Expression of TLR4/MyD88/NF-κB Insignaling in LPS-Exposed BV-2 Cells

**FIGURE 3:**
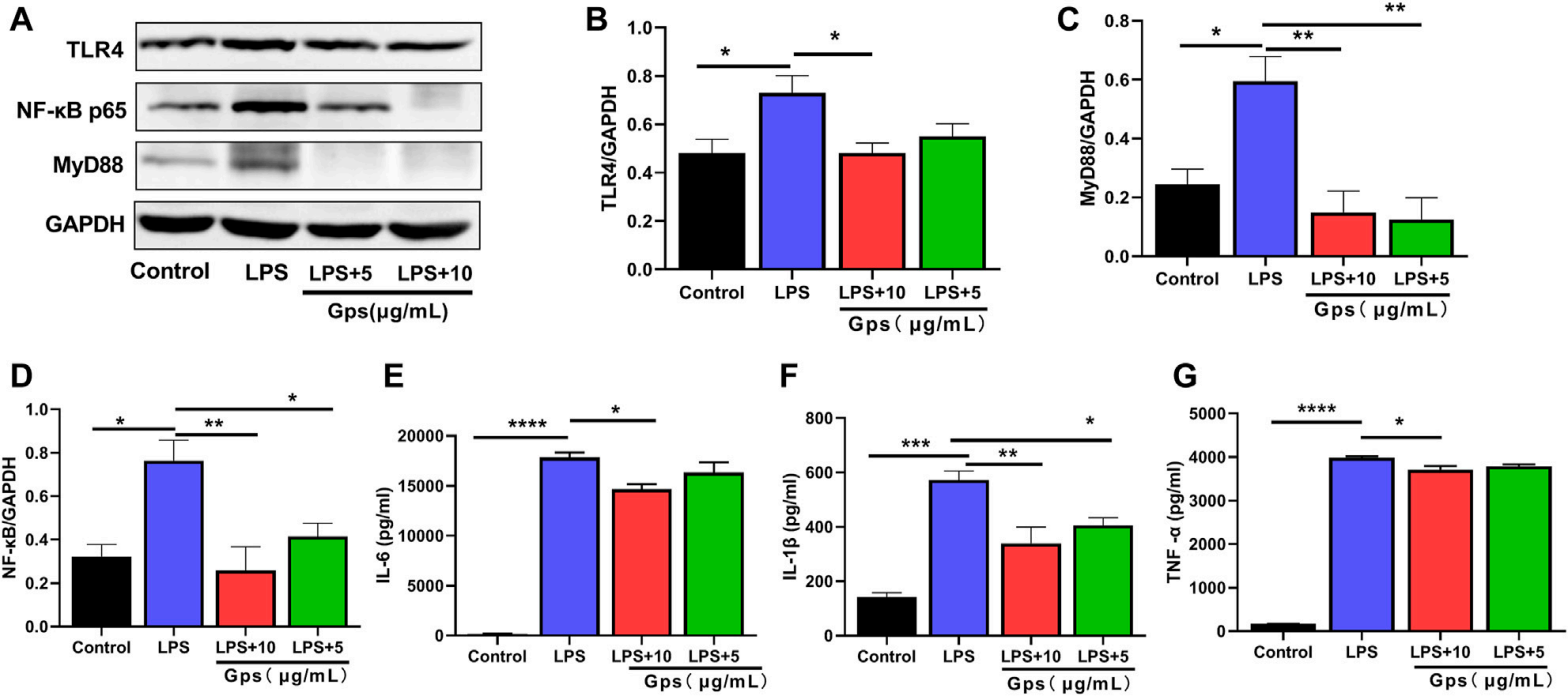
Gps inhibited the LPS-induced NF-κB signaling pathways in BV-2 cells. **(A)** Representative immunoreactive bands showing the TLR4, MyD88 and NF-κB p65 proteins in the control (untreated), LPS (50 μg/ml), LPS (50 μg/ml) and Gps (10 μg/ml or 5 μg/ml, respectively) BV-2 cells. **(B–D)** Statistical results show that LPS increased the expression of TLR4, MyD88, and NF-κB p65 proteins *in vitro*, while Gps inhibited the effects of LPS. **(E–G)** The downstream inflammatory factors IL-1β, IL-6, and TNF-α levels of TLR4/MyD88/NF-κB signaling *in vitro* were detected by ELISA. The results were expressed as mean ± SEM. *n* = 3 for each group. *, *p* < 0.05; **, *p* < 0.01; ***, *p* < 0.001; ****, *p* < 0.0001 vs. LPS.

To investigate the mechanism by which Gps shifted microglial state, we tested the expression of TLR4/MyD88/NF-κB signaling-associated proteins using western blotting or ELISA. Our data indicated that LPS exposure significantly induced the protein expression of TLR4, MyD88, and NF-κB p65 (Figures 3A–D) and markedly increased the levels of the downstream inflammatory factors IL-1β, IL-6, and TNF-α (Figures 3E–G). These findings suggest that TLR4/MyD88/NF-κB signaling is involved in LPS-induced microglial cell line state transition *in vitro*. Furthermore, Gps inhibited the effects of LPS on protein expression of TLR4, MyD88, and NF-κB p65 and the downstream inflammatory factors IL-1β, IL-6, and TNF-α (Figures 3A–G).

### 3.3 Gps Treatment Significantly Decreased Depression-like Behaviors in CUMS-Exposed Mice

**FIGURE 4:**
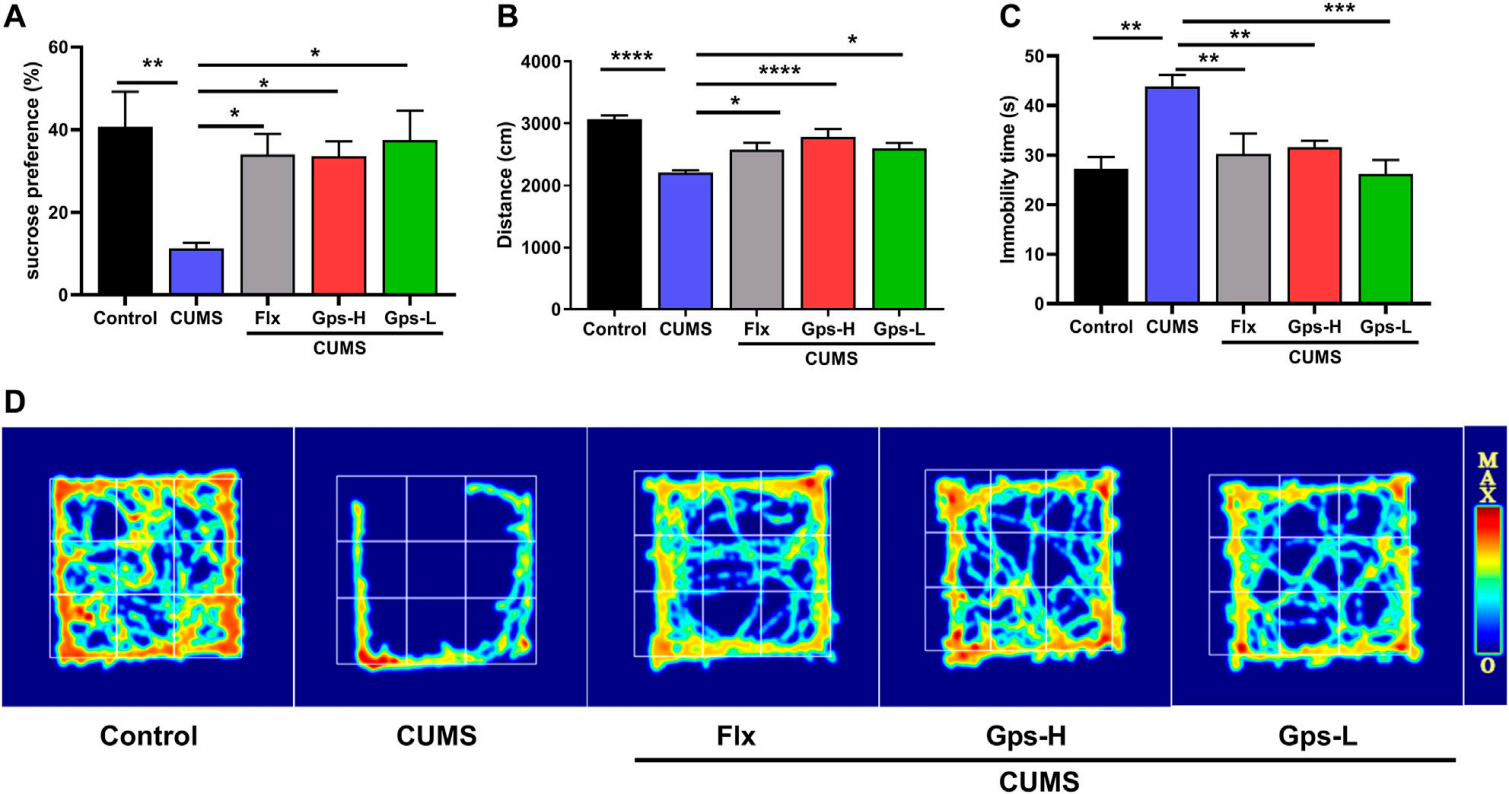
Gps blocks CUMS-induced depression-like behaviors. **(A)**. Mean sucrose preference (%), **(B)**. Movement distance in the OFT, **(C)**. Immobility time in FST, and **(D)**. The activity thermograms of mice of vehicle-administered control mice and CUMS-subjected mice administered vehicle, Flx, and high and low doses of Gps, respectively. The results were expressed as mean ± SEM. *n* = 5 for each group. *, *p* < 0.05; **, *p* < 0.01; ***, *p* < 0.001; ****, *p* < 0.0001 vs. CUMS.

To determine whether Gps can block the development of CUMS-induced depression-like behavior, we assessed depression-like behavior after treating mice with 100 or 50 mg/kg Gps. The experimental results showed CUMS reduced sucrose preference (Figure 4A), decreased OFT movement distance (Figures 4B,D), and increased FST immobility time (Figure 4C). Compared to non-CUMS control mice, the CUMS mice spent more time in the immobile position and showed a significant decrease in preference for a sucrose solution or in total moving distance. Conversely, treatment with Gps reversed these effects (Figures 4A–D) to an extent comparable with positive control drug Flx, indicating antidepressant properties of Gps.

### 3.4 Gps Treatment Promotes Monoamine Neurotransmitter Levels in CUMS-Exposed Mice

**FIGURE 5:**
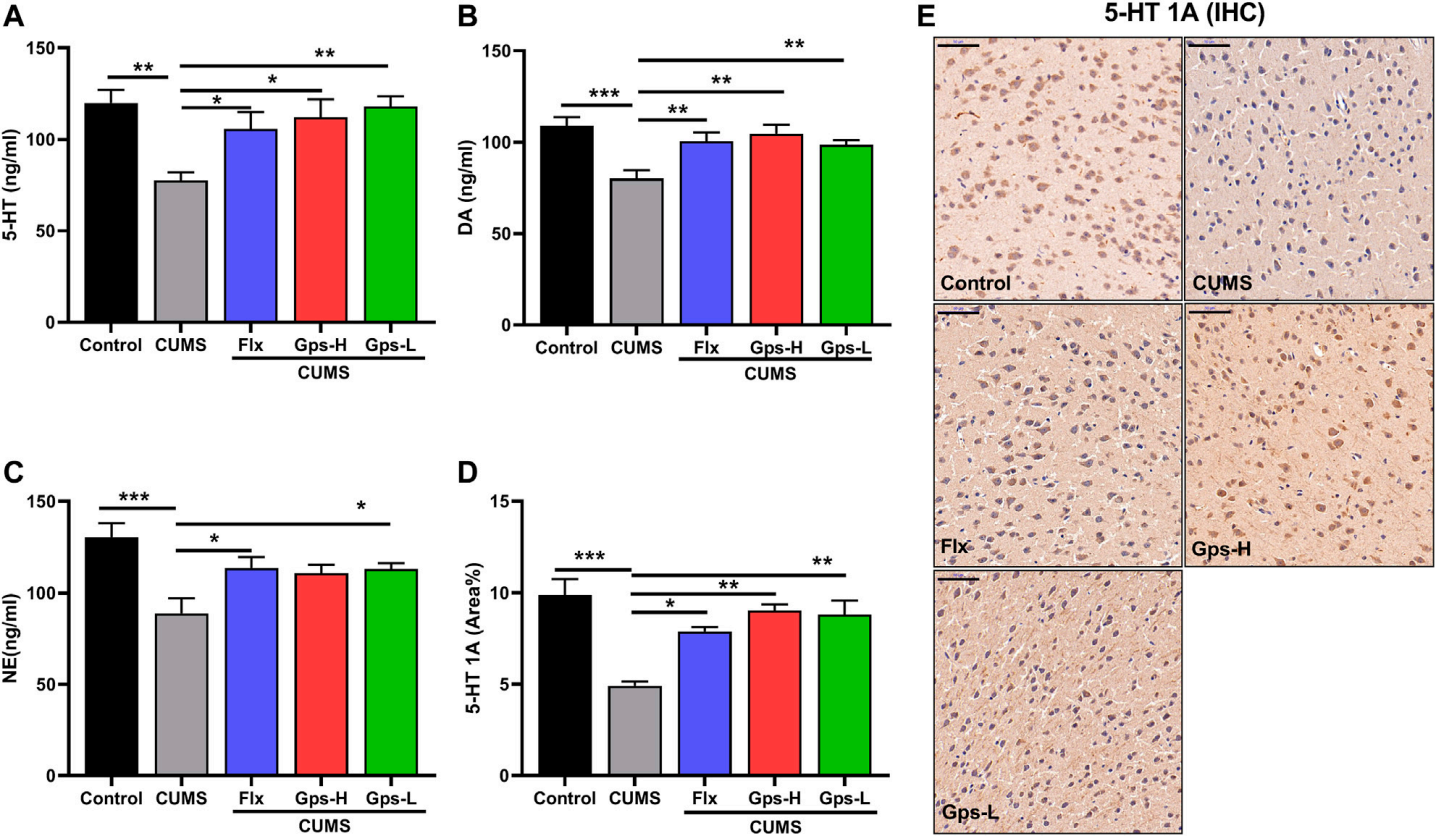
Effect of Gps on brain 5-HT, 5-HT1A, DA, and NE levels in mice with CUMS. **(A–C)** The levels of 5-HT, DA, and NE of the control (untreated), CUMS, Flx + CUMS, Gps-H + CUMS, and Gps-L + CUMS mice were determined using ELISA. *n* = 5 for each group. **(D)**. Expression of 5-HT1A in the brain was analyzed using immunohistochemistry, and the area percentage of positive staining (area %) in different groups is shown on the y-axis. *n* = 3 for each group. **(E)**. Representative immunohistochemical images of in the control (untreated), CUMS, Flx + CUMS, Gps-H + CUMS and Gps-L + CUMS mice (× 400 magnification). Scale bar indication 50 µm. The results were expressed as mean ± SEM. *, *p* < 0.05; **, *p* < 0.01; ***, *p* < 0.001 vs. CUMS.

To evaluate the antidepressant-like effects of Gps, we tested monoamine neurotransmitters in mice based on the monoamine hypothesis. As expected, we observed that CUMS led to a robust downregulation of brain 5-HT, 5-HT1A, DA, and NE levels compared with the control groups. Gps inhibited the effects of CUMS and successfully promoted an increase in monoamine neurotransmitter levels (Figures 5A–E). Moreover, this effect is equivalent to the positive control drug Flx.

### 3.5 Gps Treatment Mediates Microglial State Transition in CUMS-Exposed Mice

**FIGURE 6:**
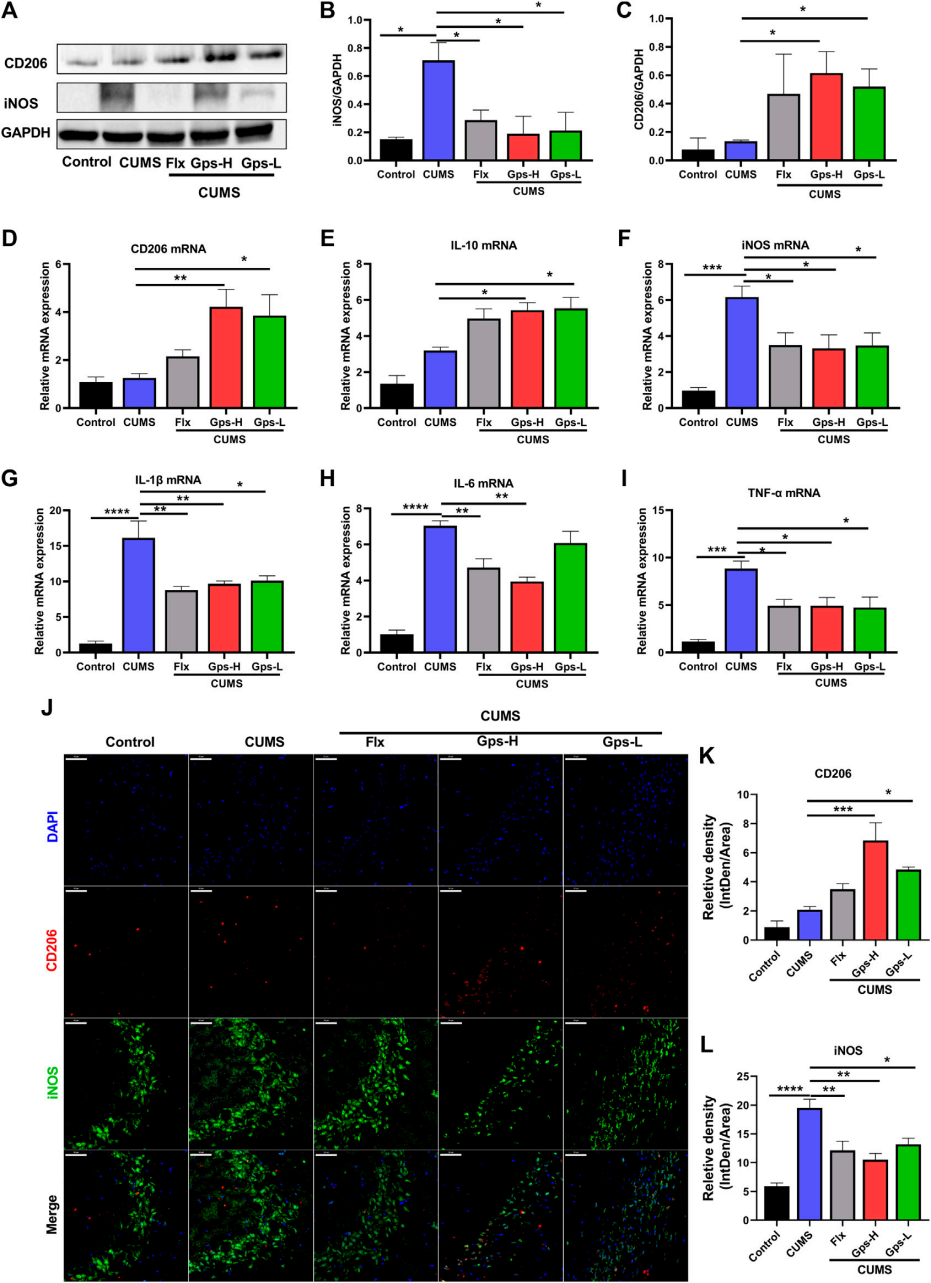
Gps regulates microglial state transition in CUMS-exposed mice. **(A)**. Representative immunoreactive bands showing the hippocampal iNOS and CD206 protein levels in the control (untreated), CUMS, Flx + CUMS, Gps-H + CUMS, and Gps-L + CUMS mice. **(B,C)** Statistical results showed that CUMS stress increased the expression of iNOS proteins in the hippocampus, while Gps inhibited the effects of CUMS and promoted the expression of CD206 protein. **(D–I)** The expression of CD206, IL-10, iNOS, IL-1β, IL-6, and TNF-α mRNA was determined using qPCR in the control (untreated), CUMS, Flx + CUMS, Gps-H + CUMS, and Gps-L + CUMS mice. **(J)**. Representative immunofluorescence images of iNOS (green) and CD206 (red) in the control (untreated), CUMS, Flx + CUMS, Gps-H + CUMS, and Gps-L + CUMS mice (× 400 magnification). Scale bar indication 50 µm. **(K,L)**. The relative fluorescence intensity of CD206 and iNOS in the control (untreated), CUMS, Flx + CUMS, Gps-H + CUMS, and Gps-L + CUMS mice. The values of iNOS and CD206 were expressed by IntDen/Area in different groups and are shown on the y-axis. The results were expressed as mean ± SEM. *n* = 3 for each group. *, *p* < 0.05; **, *p* < 0.01; ***, *p* < 0.001; ****, *p* < 0.0001 vs. CUMS.

Since depression is a microglial disease (Yirmiya et al., 2015), to determine whether CUMS-induced depression and the antidepressant effects of Gps corresponded with regulated microglial state transition, we measured the expression of inflammatory parameters and anti-inflammatory phenotype markers in the hippocampus of treated and control mice. As shown in Figure 6, we observed that CUMS increased the expression of inflammatory parameters iNOS (Figures 6A,B,F,J,L), IL-1β (Figure 6G), IL-6 (Figure 6H), and TNF-α (Figure 6I). CUMS had no statistical effect on the expression of anti-inflammatory phenotypes markers CD206 (Figures 6A,C,D,J,K) and IL-10 (Figure 6E). These data imply that alterations in microglia are associated with CUMS exposure, which in turn cause an increased vulnerability to depression-like behaviors. However, when the mice were treated with 100 mg/kg or 50 mg/kg Gps, these changes were notably attenuated as seen in western blotting (Figures 6A,B), qPCR (Figures 6F–I), and immunofluorescence (Figures 6J,L). Moreover, Gps significantly promoted the expression of anti-inflammatory phenotype markers CD206 (Figures 6A,C,D,J,K) and IL-10 (Figure 6E). Western blotting and qPCR analysis also confirmed the immunofluorescence results.

### 3.6 Gps Shifted Microglial State Transition by Inhibiting the TLR4/MyD88/NF-κB Signaling Pathway

**FIGURE 7:**
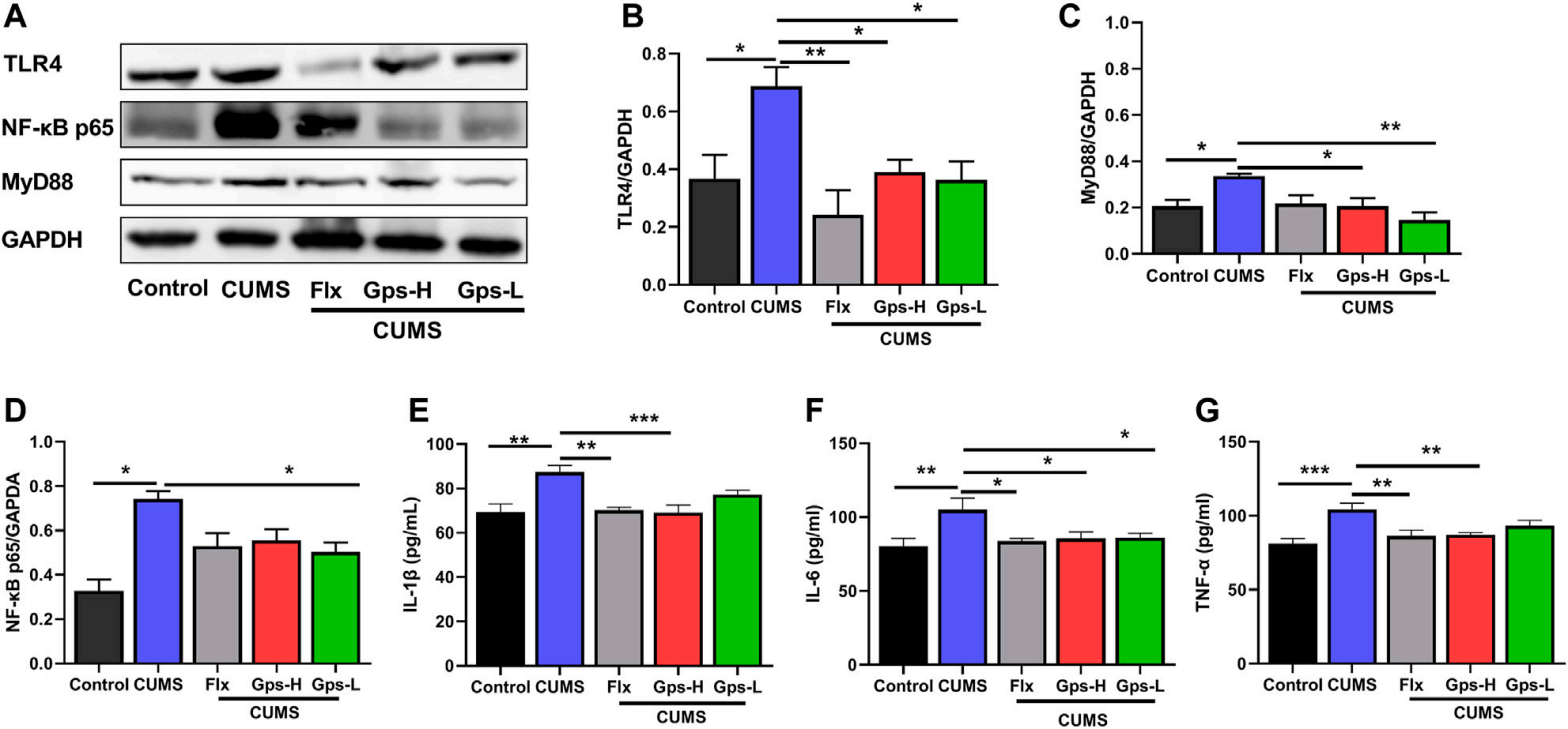
Administration of Gps regulates microglial state transition by inhibiting the TLR4/MyD88/NF-κB insignaling pathway. **(A)**. Representative immunoreactive bands showing the hippocampal proteins TLR4, MyD88, and NF-κB p65 in the control (untreated), CUMS, Flx + CUMS, Gps-H + CUMS, and Gps-L + CUMS mice. **(B–D)** Statistical results show that CUMS increased the expression of TLR4, MyD88, and NF-κB p65 proteins in the hippocampus, while Gps inhibited the effects of CUMS and suppressed the expression of TLR4, MyD88, and NF-κB p65 proteins. *n* = 3 for each group. **(E–G)** CUMS significantly promoted the levels of IL-1β, IL-6, and TNF-α, but Gps attenuated these changes. The downstream inflammatory factors IL-1β, IL-6, and TNF-α and the levels of TLR4/MyD88/NF-κB signaling were detected by ELISA. *n* = 5 for each group. The results were expressed as mean ± SEM. *, *p* < 0.05; **, *p* < 0.01; ***, *p* < 0.001 vs. CUMS.

To further determine the potential role of TLR4/MyD88/NF-κB signaling in depression, and to determine if the antidepressant-like behavior effects of Gps corresponded with a suppression in the signaling, we first established a model of depression by CUMS and treatment with Gps (100 or 50 mg/kg) or Flx. Then, the expression of hippocampal TLR4, MyD88, and NF-κB p65 were tested by western blotting. Additionally, we analyzed the levels of brain IL-1β, IL-6, and TNF-α by ELISA. Our data showed that CUMS significantly promoted the protein expression of TLR4, MyD88, and NF-κB p65 (Figures 7A–D), and also markedly promoted the levels of IL-1β, IL-6, and TNF-α (Figures 7E–G) vs control. However, when the mice were treated with 100 or 50 mg/kg Gps, these changes were notably attenuated, including downregulated protein expression of TLR4, MyD88, and NF-κB p65 (Figures 7A–D) and the downstream inflammatory factors IL-1β, IL-6, and TNF-α (Figures 7E–G). These data indicate that the mechanism of the antidepressant properties of Gps is likely through the inhibition of TLR4/MyD88/NF-κB signaling.

## 4 Discussion

**FIGURE 8:**
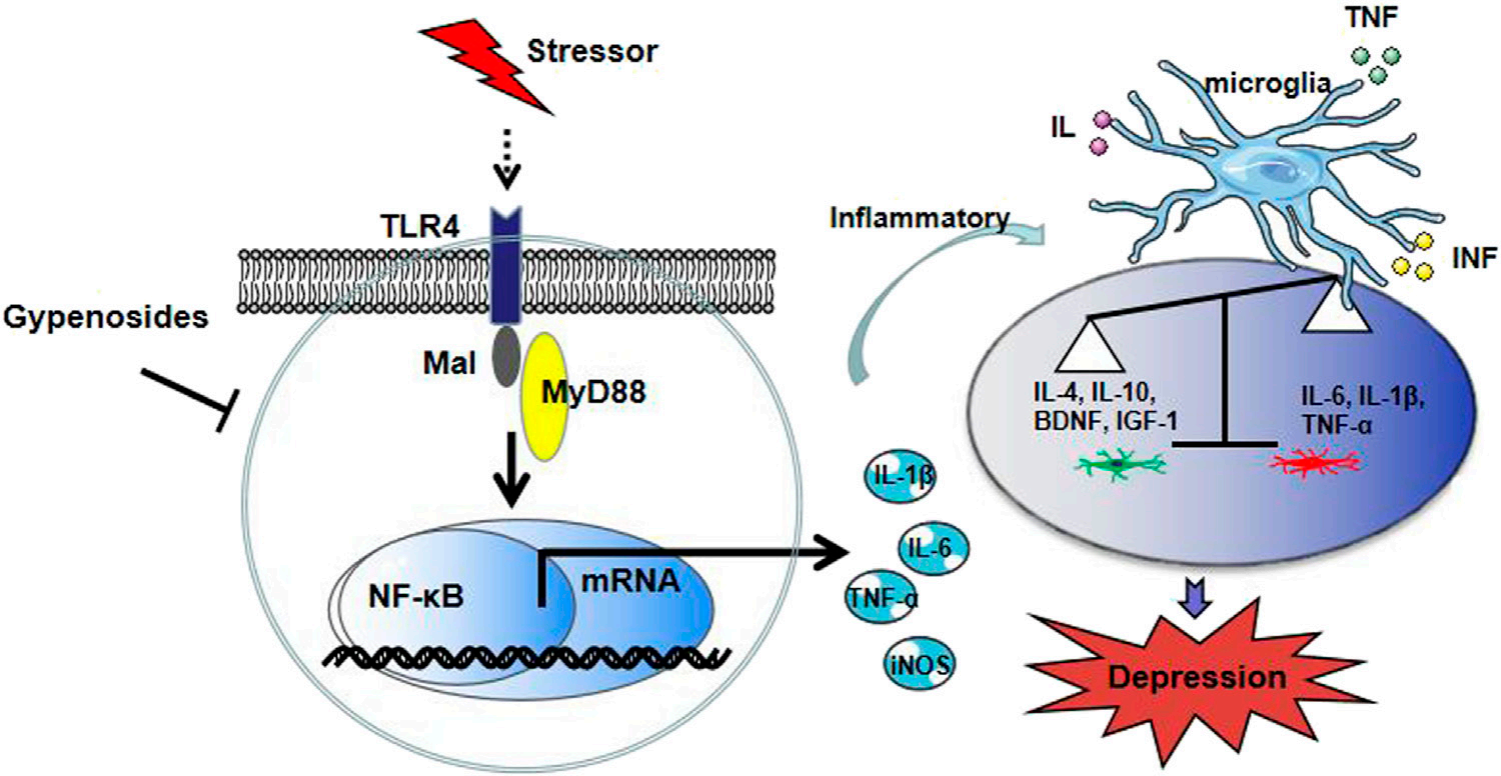
Proposed model depicting our finding on Gps blockade of depression-like behavior in mice.

In the present study, we observed that the inflammatory immune activation accompanied by microglial reactivity, and TLR4/MyD88/NF-κB signaling were activated in a CUMS-induced depression mice model and LPS-exposed BV-2 cells. The most critical finding of our *in vivo* study was that Gps improved CUMS-induced depression-like behavior through promoting microglial states into the anti-inflammatory phenotypes likely by inhibiting the TLR4/MyD88/NF-κB signaling pathway (Figure 8).

Immune processes play a very important role in CNS disorders and neuropsychiatric disorders including depressive disorders, autism spectrum disorder, and bipolar disorder (Pape et al., 2019; Song et al., 2020). Along with neurons, microglia are resident macrophages of the CNS and are a key component of the CNS immune response (Singhal et al., 2014). It has been proposed that the pathogenesis of depression has been related to the CNS inflammation caused by the activation of microglia in the brain (Debnath et al., 2021; Hayley et al., 2021). Structural and functional impairments of microglia caused by intense inflammatory activation can lead to depression (Yirmiya et al., 2015; Kalkman and Feuerbach, 2016).

The reactivity and density of microglia in brain areas (prefrontal cortex, anterior cingulate cortex, and hippocampus) are increased in patients with depression (Yang et al., 2020; Brisch et al., 2021). Indeed, changes in microglial state have been observed in different models of depression. Studies have found that exposure to CUMS potentiates the microglial proinflammatory response, contributing to development of depression-like behaviors (Tang et al., 2018; Xu et al., 2020b; Zhang et al., 2021). Injecting minocycline (microglia reactivity inhibitor) into the CUMS-induced model can significantly reduce the depression-like behavior of animals by rescuing decrease in neurogenesis in dorsal hippocampus (Bassett et al., 2021). These findings suggest that microglia in the brain undergo morphological and functional changes following stress exposure, which are considered a key of this stress-induced phenomenon. Emerging evidence indicates that regulation of microglial states transition is a possible treatment for depression disorder (Zhang et al., 2018; Prowse and Hayley, 2021). Chronic treatment with the anti-depressant ameliorated depression-like behaviors and restored microglia morphology (Xu et al.,. 2020a; Zhang et al., 2021). Xu et al. found that arctigenin modulated brain microglial phenotype, and then attenuated CUMS-induced depression (Xu et al,. 2020b). Similarly, treatment with FGF21 improved LPS-induced depression-like behavior in mice by inhibiting the inflammatory response in microglia (Wang et al., 2020).

To investigate the antidepressant properties of Gps, we established a depression model by CUMS and treatment with Gps or Flx. Then, the depressive-like behaviors of mice were determined by the OFT, FST and SPT. We found that behavioral changes were accompanied by a change in microglial states *in vivo*, consistent with previous results (Bassett et al., 2021). In parallel, Gps reversed depression-like behaviors and inhibited CNS inflammation in mice caused by CUMS as well as the expression of the inflammatory parameters TNF-α, IL-1β, IL-6, and iNOS. Interestingly, when treated with Gps, induction of the anti-inflammatory microglial phenotype markers IL-10 and CD206 was observed. In addition, the positive drug Flx inhibited the levels of inflammatory parameters iNOS, IL-1β, IL-6, and TNF-α, but there were no obvious differences in the expression of the alternative anti-inflammatory microglial phenotypes markers (IL-10 and CD206) between CUMS mice and treatment with Flx mice. *In vitro*, we found that LPS treatment induced inflammatory parameter expression but not CD206 and IL-10. The *in vivo* results were confirmed by *in vitro* data which revealed that Gps exerted a neuroprotective effect on inflammatory response in LPS-exposed BV-2 cells by inhibiting regulation of microglial cell line state transition. Together, our data demonstrated that the antidepressant properties of Gps, mediated by shifting microglial state transition *in vivo* and vitro, may be an effective antidepressant therapy. Previous studies showed that male mice demonstrated greater susceptibility to CUMS than female mice, and functional alterations in microglia were more pronounced in male mice than female mice (Wohleb et al., 2018). We therefore focused on male mice in this study. However, It is not entirely clear why male mice are more susceptive to CUMS, which require further investigation in the future.

Due to their heterogeneity, microglia have many states, and exhibit widely differing functions such as neurogenesis, neuronal circuit shaping, vascular formation, and remodeling in health and disease through multiple phenotypic changes (Stratoulias et al., 2019; Nguyen et al., 2020; Klawonn et al., 2021). Dysfunctional microglia are inextricably intertwined in depression disorders. Wohleb et al found that stress-induced microglia-mediated neuronal remodeling in the PFC contributed to synaptic deficits and development of depressive-like behavior (Wohleb et al., 2018). We described a stepwise microglial functional state transition induced by CUMS or Gps treatment, and these state transitions are responsible for the balance of pro- and anti-inflammatory mediators. Our data suggest that Gps are important modulators of microglial inflammatory responses. However, further studies are needed to assess whether Gps can affect the other microglial functions such as synaptic remodeling via shifting microglial state transition.

Furthermore, it has been reported and summarized that TLR4/MyD88/NF-κB signaling plays an important role in the aberrantly activated microglia (Feng et al., 2020). Peroxiredoxin 2 (a DAMP) interacts with TLR4 on microglia and then activates microglia through the TLR4/MyD88/NF-κB signaling pathway (Lu et al., 2018a). Among Toll-like receptors (TLRs), TLR4 is one of the recognition receptors distributed on the cell membrane and expressed on the surface of immune cell epithelial cells, including microglia. TLR4, a major receptor of LPS, can recognize and bind to CD14-MD-2-LPS and induce microglial reactivity and inflammatory responses (Qu et al., 2021). Moreover, NF-κB is one of the transcription factors implicated in TLR4 signaling that has been well-established. NF-κB signaling is activated through the TLR4-MyD88-dependent pathway, which increases the synthesis of proinflammatory factors TNF-α, IL-1β, and IL-6, resulting in the activation of the immune response in the CNS (Zusso et al., 2019). CUMS activates the TLR4 signaling (Xu et al., 2021), induces microglial reactivity, and promotes iNOS and proinflammatory cytokines TNF-α, IL-1β, and IL-6 expression in the brains of mice (Lu et al., 2019). These changes exacerbate depressive-like behaviors. In MDD patients, more intense expression of the TLR4/NF-κB pathway has been confirmed (Sales et al., 2021). Several studies have suggested that natural compounds targeting TLR4 and microglia may be developed into effective drugs or preventive strategies for the treatment of neurological diseases (Rahimifard et al., 2017).

In the present study, we observed for the first time that Gps contributes to attenuated TLR4/MyD88/NF-κB signaling in LPS-activated BV-2 cells or CUMS-exposed mice as evidenced by the protein expression of TLR4, MyD88, and NF-κB p65 and the IL-1β, IL-6, and TNF-α levels. These results indicate that Gps may regulate microglia-mediated CNS inflammation via TLR4/MyD88/NF-κB signaling. Furthermore, these results are consistent with other studies showing that TLR4/MyD88/NF-κB signaling was involved in microglial polarization induced by LPS or CUMS (Lu et al., 2018b; Feng et al., 2020). However, the signaling pathway that activates microglial polarization needs to be deciphered in further studies. In addition, There was no dose-dependent difference between the two Gps doses in the different parameters evaluated. It is possible the two range is not wide enough. It is also possible that the low dose is just as effective *in vivo* as the higher dose. Future studies will focus on the pharmacokinetics and pharmacodynamics of Gps.

Furthermore, it is important to address the root cause of a condition rather than simply mask symptoms and permit a stressor to continue damaging the body elsewhere. For instances in which inflammatory microglial phenotypes are the only harmful process, Gps supplementation may be enough for prevention or resolution of depression symptoms. For instances in which other processes are involved as well in causing the depression symptoms, Gps supplementation may still prove helpful. This is because reducing depression symptoms alone may sometimes not be enough to improve health, but may improve mood and provide motivation to pursue additional modalities. Such modalities could include adherence to a protocol to ameliorate environmental chemicals or health coaching which could help a person ameliorate initial stressors such as problematic foods.

## 5 Conclusion

The present study demonstrated that the inflammatory process driven by microglial reactivity plays a critical role in CUMS-induced depression-like behavior. Gps improved CUMS-induced depression-like behavior and exerted considerable neuroprotective effects by regulating the microglial state transition by inhibiting the TLR4/MyD88/NF-κB signaling pathway. These findings suggest that Gps is a potential antidepressant agent.

## Data Availability

The original contributions presented in the study are included in the article/Supplementary Material, further inquiries can be directed to the corresponding authors.
